# Ceftolozane/Tazobactam for the Treatment of Complicated Infections in Hospital Settings—A French Real-world Study

**DOI:** 10.1093/ofid/ofae037

**Published:** 2024-02-22

**Authors:** Jean-François Timsit, Joy Mootien, Brune Akrich, Xavier Bourge, Isabelle Brassac, Bernard Castan, Carole Mackosso, Linsay Monteiro Tavares, Fabrice Ruiz, David Boutoille, Raymond Ruimy

**Affiliations:** Réanimation Médicale et Infectieuse, AP-HP Bichat, Paris, France; Unité Fonctionnelle de Conseil en Antibiothérapie, CHU Mulhouse, Mulhouse, France; MSD France, Puteaux, France; MSD France, Puteaux, France; MSD France, Puteaux, France; Service de Médecine Interne et Maladies Infectieuses, CH Périgueux, Périgueux, France; MSD France, Puteaux, France; ClinSearch, Malakoff, France; ClinSearch, Malakoff, France; Service des Maladies Infectieuses, Nantes Université, CIC 1413, Inserm, Nantes, France; Laboratoire Médicale, CHU Nice, Nice

**Keywords:** ceftolozane-tazobactam, France, infectious disease, pneumonia, real-world evidence

## Abstract

**Background:**

This study describes the conditions of use of ceftolozane/tazobactam (C/T) and associated outcomes in French hospital settings.

**Methods:**

This was a prospective, multicenter, French observational study. Patients who received at least 1 dose of C/T were included and followed up as per routine clinical practice, until stop of C/T.

**Results:**

A total of 260 patients were enrolled between October 2018 and December 2019 in 30 centers across France. Of these, 177 (68.0%) received C/T as per indication of usage following the results of the antibiogram (documented cases). Among documented patients, the mean age was 61.8 years, 73.4% were males, and 93.8% presented with multidrug-resistant (MDR) bacteria at inclusion. C/T was most frequently prescribed for pneumonia (48.6%), bacteremia (14.7%), complicated intra-abdominal infections (13.0%), or complicated urinary tract infections (9.6%). *Pseudomonas aeruginosa* was the species most frequently isolated with 212 strains from 155 patients, and 96.2% of these strains were susceptible to C/T. The median duration of C/T treatment was 16.1 days (1–115, n = 176). Complete or partial cure was achieved in 71.7% of patients, C/T was discontinued upon adaptation to microbiology results in 11.3% of patients for the following reasons: treatment failure in 2.8%, death in 4.0%, adverse events in 1.7%, and other in 8.5%.

**Conclusions:**

This is the first prospective observational study of C/T utilization in a health care setting enrolling many patients in France. C/T demonstrated a high rate of clinical effectiveness in MDR infections, confirming it as an effective treatment option for complicated infections in a high-risk population.

The steady increase in the infections caused by multidrug-resistant (MDR) bacteria has become a major public health concern [1], including in France. MDR gram-negative pathogens are responsible for hospital-acquired pneumonia (HAP), complicated intra-abdominal infections (cIAIs), and urinary tract infections (cUTIs), including acute pyelonephritis (PN). As a result, they are associated with significant morbidity and mortality, as well as increased health care costs [2].

In response to the resistance surge to cephalosporins and fluoroquinolones, carbapenem use has increased for infections caused by MDR Enterobacteriaceae, including those producing extended-spectrum ß-lactamases (ESBL) [1, 2]. Consequently, Enterobacteriaceae have started producing carbapenemase, a ß-lactamase enzyme capable of hydrolyzing ß-lactams, mostly carbapenem. Antibiotic stewardship programs have been set up, including policies put in place by the French health authorities to monitor and reduce carbapenem use [3].

Ceftolozane/tazobactam (C/T) is a novel cephalosporin combined with a β-lactamase inhibitor, developed in response to the increasing prevalence of antibiotic resistance in gram-negative pathogens. C/T demonstrated in vitro activity against several gram-negative pathogens, including MDR *Pseudomonas aeruginosa* and ESBL-producing Enterobacteriaceae strains [4, 5]. Current indications for C/T therapy include cIAI, cUTI, acute PN, and HAP (including ventilator-associated pneumonia [VAP]) [6]. The current authorized dosing for C/T is 3 g/1.5 g per day for cIAI and cUTI and 6 g/3 g per day for pneumonia. C/T should be administered intravenously as a 1-hour infusion for all indications [6]. C/T obtained reimbursement by social insurance in France on July 26, 2016, for the treatment of cIAI, cUTI, and acute PN in adult patients [7]. Market authorization for HAP and VAP in adult patients was obtained on March 3, 2020 [6, 8].

Phase 3, prospective, randomized, double-blind trials for the treatment of cIAI (NCT01445665, NCT01445678) and cUTI (NCT01345929, NCT01345955) have reported the safety and therapeutic efficacy of C/T [9, 10]. The results showed the noninferiority of C/T therapy vs meropenem in cIAI patients and improved outcomes in cUTI patients compared with levofloxacin therapy [9, 10]. The efficacy and safety of C/T for the treatment of HAP, VAP ,or ventilated nosocomial pneumonia were evaluated in another phase 3, multicentric, prospective, double-blind randomized trial vs meropenem (NCT02070757). Study results demonstrated noninferiority of C/T compared with meropenem for treating nosocomial pneumonia [11].

Research on real-world C/T use for MDR infections has been published previously in the form of case reports, case series, and retrospective observational studies [12–18]. A real-world study conducted by Jorgensen et al. reported that C/T is effective for the treatment of complicated MDR infections in patients with limited therapeutic options [12]. However, there is need for additional real-world evidence concerning the use of C/T for complicated infections in France. This article presents the results from the CONDUCT study: Conditions of Post marketing Use of Ceftolozane/Tazobactam (ZERBAXA) in Real-World Settings: A French Multicenter Prospective Observational Study. This postauthorization study was requested by the Transparency Commission of the French National Authority for Health (HAS) to evaluate C/T use in real-world settings.

The objective of this study was to describe the demographic and clinical characteristics, susceptibility, and resistance patterns of clinical bacterial isolates to C/T and the real-world treatment patterns and outcomes of patients treated with C/T.

## METHODS

### Study Design and Population

CONDUCT was a prospective, multicenter observational study across 28 sites in France. Consecutive patients who had received at least 1 dose of C/T were included in a registry between 11/10/2018 and 30/12/2019. Each patient was followed up until stop of C/T (last dose received). The total study duration, including the observational period for all study patients, was ∼17 months.

As C/T can only be prescribed in the hospital, the study setting was limited to hospitals (both public and private) and included all hospital wards and intensive care units responsible for the management of infections. A registry was maintained to collect data regarding all C/T prescriptions to control for potential selection bias. Therefore, participating centers were collecting data about C/T prescription, and then corresponding patients were solicited for their consent to be part of the study. All patients were treated as per the standard of care at each site, and treatment decisions were at the physician’s discretion.

### Variables and Measurements

Relevant demographic, clinical, laboratory, and infection characteristics, C/T prescription, and prior and concomitant treatment data were collected using an electronic case report form. The Charlson Comorbidity Index (CCI), which predicts 10-year survival in patients with multiple comorbidities, was calculated for all patients [19]. Severity was assessed using the Sequential (Sepsis-related) Organ Failure Assessment (SOFA) score [20]. Patients with a SOFA score of 6 or higher were classified as severely ill. Information concerning risk factors for MDR infection was collected as per the French Society of Anesthesia and Intensive Care Medicine (SFAR) guidelines. MDR was defined as acquired nonsusceptibility to at least 1 agent in 3 or more antimicrobial categories [21].

Outcomes included: complete cure—not requiring therapy escalation or additional antibacterial therapy or resolution of the index infection; partial cure—partial resolution of clinical signs and symptoms and/or additional antibiotic therapy at the end of C/T therapy; and treatment failure—any situation requiring a change in antibacterial therapy for the index infection due to documented lack of clinical response, need for therapy escalation, a positive culture reported at the end of C/T treatment, the need for reoperation for source control, or death while receiving C/T. Any antibiotic started before or on the day of C/T initiation and administered for at least 2 days along with C/T was considered concomitant treatment. The position of C/T in therapy was described with respect to prior antibiotic and concomitant antibiotic therapy and following therapeutic lines. Microbiology results concerning bacterial susceptibility to C/T were analyzed by a central laboratory.

### Statistical Analysis

All objectives were analyzed using standard descriptive statistics. Continuous variables were summarized with number, mean, standard deviation, median, interquartile range (IQR), minimum and maximum, and number of missing data, where applicable. Categorical variables were summarized with frequencies, percentages, and number of missing data. Missing data were considered missing without any imputation. SAS statistical software (version 9.4; SAS Institute Inc., Cary, NC, USA) was used for all analyses.

### Ethics

CONDUCT was approved by the French Ethics Committee (CCTIRS) on May 14, 2018, (application number 2018-A00308-47). The protocol was approved by the French Informatics Commission (CNIL) on February 3, 2017 (application number 2031803).

## RESULTS

A total of 260 patients among 432 patients entered in the registry were enrolled in the study. Of note, among the patients who were in the registry but were not included in the study (n = 174), there was a significantly greater proportion of patients aged >65 years and with cUTI compared with the enrolled group. A detailed analysis of the registry is presented in Supplementary Table 1. Reasons for noninclusion as reported by the investigating centers are listed in Supplementary Table 2.

Out of 260 enrolled patients, 177 (68.0%) received C/T following the results of the antibiogram. The remaining 83 patients were treated empirically; their main clinical baseline data as well as outcomes are reported in Supplementary Table 3. We will focus the description on patients who were treated following the result of the antibiogram (documented patients).

The baseline demographic and clinical characteristics of documented patients, the MDR subgroup, and the severely ill subgroups are presented in Table 1. The mean age (SD) was 61.8 (17.0) years, and most patients (73.4%) were male. The most frequent comorbidities were chronic pulmonary disease (32.8%), diabetes (27.1%), and renal failure (21.5%). In total, 32.8% of patients were immunocompromised (n = 58/177; ie, with ongoing immunosuppressive therapy or with ongoing chemo-/radio-/corticotherapy, or with hemopathy, metastatic cancer, or HIV+ with CD4 lymphocytes <500/mm^3^ at inclusion). In a majority of patients (n = 166), MDR isolates were linked to the infection, and 25.6% (n = 41/160) were classified as severely ill (SOFA score ≥6) at inclusion (Table 1). Risk factors for MDR were reported for 129 patients. In total, 46 patients (35.7%) had 1 risk factor for MDR infection, and 83 presented with >1 risk factor (64.3%). The most common risk factors for MDR infection were being a carrier of an ESBL-positive Enterobacteriaceae or ceftazidime-resistant *P. aeruginosa* identified on microbiology in the last 3 months, previous treatment with a third-generation cephalosporin or fluoroquinolone in the past 3 months, and treatment failure with broad-spectrum antibiotic therapy with third-generation cephalosporin or fluroquinolone or piperacillin/tazobactam (Table 1).

**Table 1.** Baseline Demographic and Clinical Characteristics of Documented Patients, the MDR Subgroup, and the Severely Ill Subgroup

	Documented Patients (n = 177)^a^	MDR Subgroup (n = 166)^a^	Severely Ill (SOFA Score ≥6) Subgroup (n = 41)^a^
Age, mean (SD), y	61.8 (17.0)	61.5 (17.3)	65.1 (14.4)
Male sex	130 (73.4)	121 (72.9)	34 (82.9)
BMI, mean (SD), kg/m^2^	25.5 (6.4)	25.6 (6.5)	28.3 (6.5)
Underweight [<18.5]	23 (13.5)	21 (13.1)	2 (5.0)
Overweight [≥25 to 30]	46 (26.9)	42 (26.3)	14 (35.0)
Obese [>30]	35 (20.5)	34 (21.3)	13 (32.5)
Patients with medical or surgical history	153 (86.4)	143 (86.1)	36 (87.8)
Patients with >1 medical/surgical antecedent	93 (60.8)	89 (62.2)	19 (52.8)
Most frequent comorbidities	…	…	…
Chronic pulmonary pathology	58 (32.8)	54 (32.5)	10 (24.4)
Diabetes	48 (27.1)	47 (28.3)	12 (29.3)
Renal failure	38 (21.5)	36 (21.7)	12 (29.3)
Tumor without metastases [tumors diagnosed >5 y before inclusion were excluded]	29 (16.4)	27 (16.3)	8 (19.5)
Myocardial infarction	17 (9.6)	17 (10.2)	4 (9.8)
Congestive heart failure	13 (7.3)	13 (7.8)	2 (4.9)
Immunocompromised patients	58 (32.8)	54 (32.5)	15 (36.6)
Charlson comorbidity index ≥5	85 (48.0)	80 (48.2)	25 (61.0)
Baseline creatinine clearance, mL/min	n = 169	n = 158	n = 37
Glomerular hyperfiltration >150	21 (12.4)	19 (12.0)	1 (2.7)
50–150	101 (59.8)	96 (60.8)	14 (37.8)
30–50	20 (11.8)	17 (10.8)	12 (32.4)
15–30	18 (10.7)	17 (10.8)	6 (16.2)
Severe renal impairment <15	9 (5.3)	9 (5.7)	4 (10.8)
Risk factors for MDR infection for patients with only 1 risk factor	n = 129	n = 121	n = 28
Carrier of an ESBL-positive enterobacterium or ceftazidime-resistant *P. aeruginosa* on a sample <3 mo old	19 (41.3)	19 (42.2)	2 (25.0)
Previous treatment with a third-generation cephalosporin or fluroquinolone in the past 3 mo	17 (37.0)	16 (35.6)	4 (50.0)
Treatment failure with broad-spectrum antibiotic therapy with third-generation cephalosporin or fluroquinolone or piperacillin/tazobactam	6 (13.0)	6 (13.3)	1 (12.5)
Early recurrence [<15 d] of an infection treated with piperacillin-tazobactam for at least 3 d	2 (4.3)	2 (4.4)	1 (12.5)
Patient living in a nursing home or long-term care with an indwelling catheter and/or a gastrostomy	2 (4.3)	2 (4.4)	-

Abbreviations: BMI, body mass index; ESBL, extended-spectrum ß-lactamase; MDR, multidrug-resistant; SOFA, Sequential (Sepsis-related) Organ Failure Assessment score.

^a^Unless otherwise specified, values refer to No. (%).

**Figure 1.**
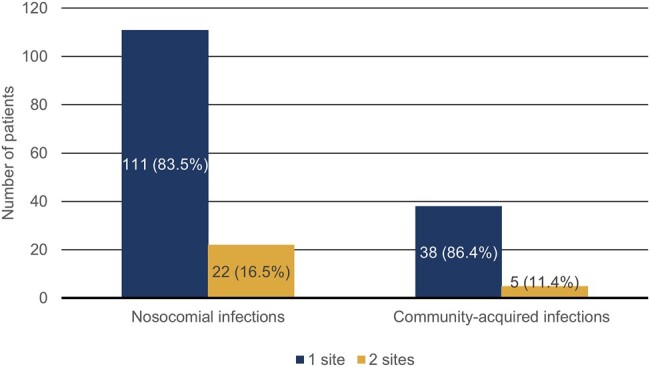
Number of infection sites at baseline for patients with nosocomial and community-acquired index infections in the documented population. Only 1 patient with a community-acquired infection presented with 3 infection sites at baseline.

Among patients who received treatment after documentation, 133 patients (75.1%) had a reported nosocomial index infection, while 44 patients (24.9%) had a community-acquired index infection. Of all patients, 15.8% (n = 28/177) were reported with 2 or more infection sites. Figure 1 reports the number of infection sites at baseline (1 or 2 sites) for patients with community-acquired and nosocomial infections.

In total, 48.6% (n = 86) of patients had a diagnosis of pneumonia. Furthermore, 64 (74.4%) pneumonia infections were hospital-acquired, and a total of 43 pneumonia patients required mechanical ventilation. Bacteremia, cIAI, cUTI, skin and soft tissue infection, and acute PN were less frequently reported (respectively: 14.7%, 13.0%, 9.6%, 7.3%, and 5.6%). Catheter infection and sepsis of undetermined origin were reported in 2.8% and 0.6%, respectively. Table 2 reports the indications for all infection sites.

**Table 2.** Description of C/T Indications at Baseline for Documented Patients—All Infection Sites

	Documented Patients (n = 177)	Nosocomial Infection Subgroup (n = 133)	Community-Acquired Infection Subgroup (n = 44)
All infection sites^a^	…	…	…
No.	200	153	47
cIAI	23 (13.0)	19 (14.3)	4 (9.1)
cUTI	17 (9.6)	13 (9.8)	4 (9.1)
Acute PN	10 (5.6)	6 (4.5)	4 (9.1)
Pneumonia	86 (48.6)	64 (48.1)	22 (50.0)
Bacteremia	26 (14.7)	23 (17.3)	3 (6.8)
Skin and soft tissue infection	13 (7.3)	10 (7.5)	3 (6.8)
Catheter infection	5 (2.8)	4 (3.0)	1 (2.3)
Sepsis of undetermined origin	1 (0.6)	1 (0.8)	-
Other	19 (10.7)	13 (9.8)	6 (13.6)
Bone infection^b^	4 (21.1)	3 (23.1)	1 (16.7)
Osteoarticular infection^b^	4 (21.1)	3 (23.1)	1 (16.7)
Other respiratory infection^b^	3 (15.8)	…	3 (50.0)
Not specified/other^b^	8 (42.1)	7 (53.8)	1 (16.7)

Abbreviation: C/T, ceftolozane/tazobactam; cIAI, complicated intra-abdominal infection; cUTI, complicated urinary tract infection.

^a^Percentages were calculated using the total number of patients in each of the 3 groups.

^b^Percentages were calculated using the total number of patients in the group “other.”

### Previous Treatments

**Figure 2.**
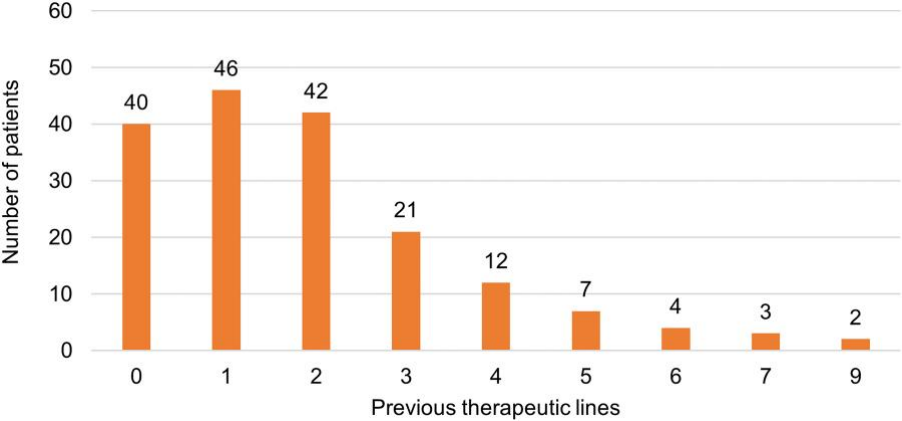
Number of therapeutic lines for the treatment of the index infection in patients with a documented prescription before C/T initiation. Abbreviation: C/T, ceftolozane/tazobactam.

For the index infection, 77.4% (n = 137/177) of patients received antibiotic treatment before C/T, of whom 91 (66.4%) received >1 therapeutic line. Distribution of the number of prior treatments is reported in Figure 2. Carbapenems were the most commonly prescribed antibiotic in the therapeutic line immediately before C/T (25.5% of patients), followed by cephalosporins (18.2% of patients) and penicillins (16.1% of patients). Stop of prior antibiotics was more often due to adaptation to microbiology results (72.3% of cases).

### Ceftolozane/Tazobactam Prescription

**Figure 3.**
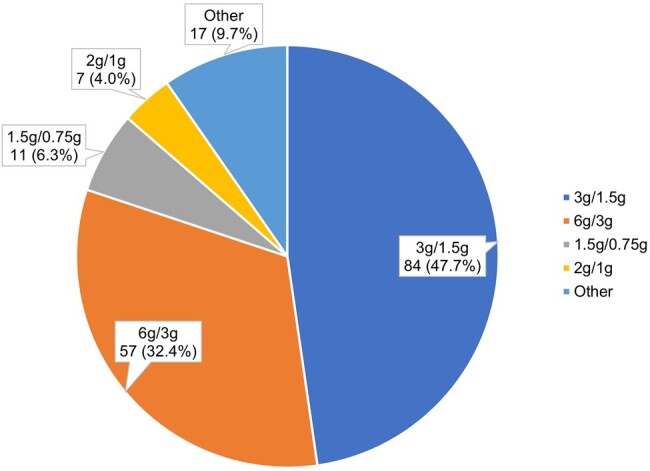
Daily dose of C/T prescribed at baseline for documented patients. The dose was missing for 1 patient. Abbreviation: C/T, ceftolozane/tazobactam.

Many of the patients receiving C/T were in the intensive care unit (ICU; 44.6%), followed by the pneumology department (11.3%). The most frequently administered daily doses were 3 g/1.5 g and 6 g/3 g, irrespective of severity of illness or higher CCI scores. Figure 3 reports the various C/T daily doses prescribed. Among patients with pneumonia and a creatinine clearance >50 mL/min, we observed a balanced distribution between total daily doses of 3 g/1.5 g or 6 g/3 g.

Among the 46 patients with impaired renal function (<50 mL/min), 21 (45.6%) patients were prescribed C/T at daily doses <3 g/1.5 g. Among the 21 patients with glomerular hyperfiltration, 11 (52.4%) received a total daily dose of 6 g/3 g. The median duration of therapy was 10 days for patients with C/T therapy. A longer median duration was observed for patients with C/T and concomitant treatments for 15 days.

### Concomitant Treatments

Almost half of the patients (40.7%, n = 72/177) received concomitant antibiotic therapy. Among patients receiving concomitant treatment, 25 patients initiated another antibiotic before C/T start, while concomitant treatment was initiated on the same day as C/T for 50 (69.4%) patients. Of the patients receiving concomitant antibiotics, 15 (21.0%) received 2 or more. Concomitant antibiotics targeting gram-negative bacteria (G– and G+/–) accounted for 73.3% (n = 66/90) of prescriptions. Among patients receiving only 1 concomitant treatment, the most frequently administered classes of antibiotics were aminoglycosides (29.8%, n = 17/57) and fluoroquinolones (21.1%, n = 12/57). The mean duration of concomitant treatment was 16.4 ± 17.5 days, with a median of 11.5 days.

### Microbiology Results and Patients With *P. aeruginosa* Isolates

In total, MDR isolates tested for susceptibility to C/T were found in 149 (84.2%) of all documented patients. *Pseudomonas aeruginosa* was the species most frequently isolated. From 138 patients, 185 strains of *P. aeruginosa* were isolated, of which 96.8% were susceptible to C/T. Table 3 reports the microbiology results in detail.

**Table 3.** Microbiology Results—MDR Bacteria Isolated From Documented Patients Tested for Susceptibility to C/T

	No. of Patients (n = 149)	No. of Strains (n = 235)	Susceptibility to C/T (n = 221)	Resistance to C/T (n = 14)
Bacteria				
*Citrobacter braakii*	1	1	1 (100)	-
*Citrobacter freundii*	1	1	1 (100)	-
*Citrobacter koseri*	1	1	1 (100)	-
*Enterobacter aerogenes*	1	1	-	1 (100)
*Enterobacter cloacae*	5	5	5 (100)	-
*Escherichia coli*	11	12	12 (100)	-
*Klebsiella aerogenes*	1	1	-	1 (100)
*Klebsiella oxytoca*	1	1	1 (100)	-
*Klebsiella pneumoniae*	17	23	19 (82.6)	4 (17.4)
*Klebsiella variicola*	1	1	1 (100)	-
*Pseudomonas aeruginosa*	138	185	179 (96.8)	6 (3.2)
*Serratia marcescens*	2	2	1 (50)	1 (50)
*Stenotrophomonas maltophilia*	1	1	-	1 (100)

Abbreviations: C/T, ceftolozane/tazobactam; MDR, multidrug-resistant.

At inclusion, 90.4% of patients presented with *P. aeruginosa* (n = 160/177) isolates. Of these, 141 had at least 1 medical antecedent, with chronic pulmonary disease (n = 56), diabetes (n = 47), and renal failure (n = 37) being the most frequent ones. The majority were diagnosed with pneumonia (51.3%, n = 82), 21 with cIAI (13.1%), 19 with bacteraemia (11.9%), and 14 with cUTI (8.8%). Most patients received 1 or more treatments before C/T to treat the index infection (76.9%, n = 123/160), with carbapenems being the most frequently prescribed immediately before C/T (22.8%). The majority of patients received either the 3 g/1.5 g (48.4%, n = 77) or the 6 g/3 g (32.1%, n = 51) C/T daily doses, irrespective of type of index infection. In total, 40.7% of documented *P. aeruginosa* patients received at least 1 concomitant treatment (n = 68): 23 (33.8%) were initiated before start of C/T, while 48 (70.6%) were initiated on the same day as C/T. The mean duration of concomitant treatment (SD) for this subgroup was 15.8 (17.6) days.

### Outcomes (Reasons for Stopping C/T)

Patient outcomes are reported in Table 4. More than two-thirds of all patients stopped C/T thanks to complete cure (63.8%, n = 113/177) or partial cure (7.9%, n = 14/177). Therapy de-escalation following microbiology results was the main reason for C/T discontinuation (11.3%, n = 20/177). C/T was stopped due to treatment failure in 5 (2.8%) patients and due to occurrence of an adverse event (AE) in 3 (1.7%) patients. Of the 177 patients with documented prescriptions, 113 (63.8%) were reported completely cured, while in patients with *P. aeruginosa* isolates the percentage was slightly higher, with 66.3% (n = 106/160); adaptation to microbiology results was the reason to stop C/T in 15 (9.4%) patients, due to occurrence of an AE in 3 (1.9%) and due to treatment failure in 4 (2.5%). Other reasons were cited for 12 (7.5%) *P. aeruginosa*–infected patients. In total, 7 (4.4%) deaths were reported at the end of C/T treatment. Four (57.1%) were linked to the index infection.

**Table 4.** Description of Outcomes of Reasons for Stopping C/T for Documented Patients

Outcomes of Reasons for Stopping C/T	Documented Patients (n = 177)
Cured	113
Mean duration of treatment until stop (SD), d	18.8 (17.3)
Median	15.0
Range	1.0–115.0
Partial cure	14
Mean duration of treatment until stop (SD), d	15.0 (8.2)
Median	11.5
Range	5.0–31.0
Adaptation to microbiology results	20
Mean duration of treatment until stop (SD), d	8.4 (11.8)
Median	4.5
Range	1.0–49.0
Occurrence of an adverse event leading to stop of C/T	3
Mean duration of treatment until stop (SD), d	8.7 (4.0)
Median	8.0
Range	5.0–13.0
Treatment failure	5
Mean duration of treatment until stop (SD), d	8.3 (6.7)
Median	7.5
Range	1.0–17.0
Death	7
Mean age at death (SD), y	66.9 (23.0)
Median	72.3
Range	19.3–87.5
Mean duration of treatment (SD), d	11.3 (13.7)
Median	5.0
Range	2.0–41.0
Death linked to infection	4 (57.1)
Other reasons for stop	15
Mean duration of treatment until stop (SD), d	12.7 (12.2)
Median	9.0
Range	1.0–45.0

Abbreviation: C/T, ceftolozane/tazobactam.

## DISCUSSION

Phase 3 registration trials should always be confirmed in real-world practice. It is essential to obtain real-world evidence to ascertain the external validity of trial results in routine treatment and to demonstrate the value of the drug [6, 12, 22]. This real-world evidence study sought to add to currently available evidence by assessing the real-world treatment pattern of C/T across several tertiary hospitals in France. The diversity of tertiary hospitals included in this study was interesting given their geographical location across all France and their typology, that is, teaching university hospital, general hospital, public and private–public settings.

Most C/T prescriptions followed the indication of usage and were documented by the results of the antibiogram. This is in line with the HAS guidelines, which recommend avoidance of empirical prescriptions [3]. A majority of patients who received C/T were infected by MDR bacteria and had multiple comorbidities. Despite this, the outcomes among patients treated with C/T were positive, as 63.8% were fully cured. Susceptibility testing demonstrated that there was a high rate of susceptibility to C/T for a majority of the isolates tested, including *P. aeruginosa*. It should be noted that 96.2% of *P. aeruginosa* strains were susceptible to C/T. The elaborate microbiology testing highlights the well-organized laboratory system in place in French hospitals. In contrast to randomized controlled trials, 40.7% of patients were on concomitant antibiotic treatment, with a majority targeting gram-negative bacteria [6, 12]. C/T dosing of 3 g/1.5 g and 6 g/3 g daily was evenly distributed among patients with normal creatinine clearance. C/T dose adaptation for subjects with impaired renal function or glomerular hyperfiltration was prescribed for ∼50% of patients. For the remaining patients, dose adaptation was not strictly followed, probably due to their clinical characteristics, such as older age, CCI ≥3–4, and severe comorbidities (data not shown).

It was observed that C/T was mainly prescribed for *P. aeruginosa* infections. As seen in previous studies, C/T susceptibility was high among *P. aeruginosa* strains, with 96.8% strains demonstrating C/T susceptibility [23, 24]. As published in other studies, C/T maintains a high level of susceptibility even when MDR or difficult-to-treat resistant (DTR) strains are tested. It should also be noted that C/T’s susceptibility level makes it a drug of choice when treating *P. aeruginosa* infections, especially in case of resistance to first-line treatments [25, 26].

The rate of complete cure was 63.8%, with low mortality (4.0%, n = 7/177). Treatment failures were also low (2.8%, n = 5). Among these 5 patients, 4 presented with MDR isolates at inclusion, and only 1 developed resistant phenotypes to C/T during treatment. The low rate of development of resistance observed is aligned with observation made during the ASPECT-NP trial, in which no *Pseudomonas* strains acquired resistance during treatment [27].

Direct comparison of these results with randomized controlled trials like ASPECT is not feasible as the study design did not permit recording of clinical outcomes post–C/T stop for other reasons. Outcomes could be impacted by several factors, including concomitant antimicrobial therapy, delayed treatment, clinical management, and other unobservable factors [6]. Nonetheless, other observational studies like SPECTRA have demonstrated the same results in terms of the efficacy and indications treated [28]. A systematic literature review recently published corroborated those results, demonstrating that C/T is effective in clinical practice, despite the diverse group of seriously ill patients, different levels of resistance of the pathogens treated, and varying dosing regimens used [29].

Our study, being observational in nature, could be impacted by selection bias. We have attempted to limit this by performing consecutive inclusion of patients and the maintenance of a registry to record all C/T prescriptions made at participating sites. Moreover, although the study included only French patients (and, therefore, the generalizability of the results to other countries should be carefully taken into account according to national guidelines), the relatively large cohort provides an adequate description of patients treated with C/T in real-life clinical conditions.

## CONCLUSIONS

This real-world prospective study adds to the literature on management of complicated infections using C/T. This is the first study focusing on French patients demonstrating that C/T is mainly used for high-risk patients with complicated infections. Most patients presented with *P. aeruginosa* infections, mainly MDR, in which C/T achieved 96.8% susceptibility. Overall, the rate of success is encouraging for a real-world study, especially given the severity of included patients.

## Supplementary Material

ofae037_Supplementary_Data

## Supplementary Data

Supplementary materials are available at *Open Forum Infectious Diseases* online. Consisting of data provided by the authors to benefit the reader, the posted materials are not copyedited and are the sole responsibility of the authors, so questions or comments should be addressed to the corresponding author.
