# Supplementary material for: Ceftolozane/Tazobactam for the Treatment of Complicated Infections in Hospital Settings—A French Real-world Study
Source: Open Forum Infect Dis. 2024 Feb 22;11(2):ofae037. doi: 10.1093/ofid/ofae037 (PMC10883286; doi:10.1093/ofid/ofae037)
Supplement: ofae037_Supplementary_Data [file ofae037_supplementary_data.docx]

**Supplementary Table 1** Baseline characteristics of patients not included and included in the study

|  | Non-included patients (N=174) | Included patients (N=258)* | Total (N=432) | p-value |
| --- | --- | --- | --- | --- |
|  |  |  |  |  |
| Age groups |  |  |  | ***0.036*** |
| Missing | - | 1 (0.4) | 1 (0.2) |  |
| < 18 years | 6 (3.4) | 4 (1.6) | 10 (2.3) |  |
| 18 – 40 years | 50 (28.7) | 64 (24.8) | 114 (26.4) |  |
| 41 – 65 years | 69 (39.7) | 83 (32.2) | 152 (35.2) |  |
| > 65 years | 49 (28.2) | 106 (41.1) | 155 (35.9) |  |
|  |  |  |  |  |
| Sex, N | 173 | 257 | 430 | ***0.048*** |
| Men | 101 (58.4) | 174 (67.7) | 275 (64.0) |  |
|  |  |  |  |  |
| Indications |  |  |  |  |
| Ciai | 8 (4.6) | 22 (8.5) | 30 (6.9) | 0.115 |
| cUTI | 4 (2.3) | 24 (9.3) | 28 (6.5) | ***0.004*** |
| Acute PN | 5 (2.9) | 7 (2.7) | 12 (2.8) | 1.000 |
| Respiratory infection | 104 (59.8) | 150 (58.1) | 254 (58.8) | 0.736 |
| Bacteraemia | 30 (17.2) | 28 (10.9) | 58 (13.4) | 0.056 |
| Skin and soft tissue infection | 3 (1.7) | 6 (2.3) | 9 (2.1) | 0.745 |
| Catheter infection | 2 (1.1) | 3 (1.2) | 5 (1.2) | 1.000 |
| Sepsis of undetermined origin | 8 (4.6) | 3 (1.2) | 11 (2.5) | ***0.032*** |
| Other | 26 (14.9) | 32 (12.4) | 58 (13.4) | 0.448 |
| * 2 included patients were not reported in the registry | | | | |

**Supplementary Table 2** Description of non-included patients

|  | N (%) |
| --- | --- |
| N | 146* |
| Patient refused study participation | 10 (6.8) |
| Other reasons | 136 (93.1) |
| Study not proposed | 32 (21.9) |
| Patient already included for a precedent infection | 25 (17.1) |
| Premature stop | 16 (11.0) |
| Treatment already started | 14 (10.3) |
| Discharge before study inclusion | 12 (8.2) |
| Unfavourable clinical situation | 12 (8.2) |
| Death less than 48 hours after treatment start | 5 (3.4) |
| Change of treatment | 4 (2.7) |
| Incorrect prescription | 3 (2.1) |
| Treatment not administered | 2 (1.4) |
| Inclusion in another study | 2 (1.4) |
| Other^1^ | 9 (6.6) |
| * Reason for non-inclusion missing for 10 patients  1 “Other” reported reasons vary significantly between investigating centres |  |

**Supplementary Table 3** Description of patients with empirical prescription

|  | Empirical patients  (N=83)* |
| --- | --- |
| **Age (years), mean (SD)^1^** | 46.3 (18.3) |
| **Male Sex** | 45 (54.2) |
| **BMI (kg/m^2^), mean (SD)** | 23.0 (6.6) |
| Underweight [< 18.5] | 15 (18.3) |
| Overweight [≥ 25 to 30] | 12 (14.6) |
| Obese [> 30] | 8 (9.8) |
| **Patients with medical or surgical history** | 78 (94.0) |
| Patients > 1 medical/surgical antecedent | 44 (56.4) |
| **Immunocompromised patients** | 21 (25.3) |
| **Charlson comorbidity index ≥ 5** | 18 (21.7) |
| **Baseline creatinine clearance (mL/min)** | N=71 |
| Glomerular hyperfiltration > 150 | 6 (8.5) |
| 50 – 150 | 47 (66.2) |
| 30 – 50 | 6 (8.5) |
| 15 – 30 | 8 (11.3) |
| Severe renal impairment < 15 | 4 (5.6) |
| **All infection sites** | N=83 |
| cIAI | 3 (3.6) |
| cUTI | 5 (6.0) |
| Acute PN | 1 (1.2) |
| Pneumonia | 64 (77.1) |
| Other infection sites | 15 (18.1) |
| **Outcomes of reasons for stopping C/T** | N=83 |
| *Mean duration of treatment till stop (SD) (days)* | 14.8 (11.5) |
| *Median* | 15.0 |
| *Range* | 2.0 – 93.0 |
| **Cured** | 24 (28.9) |
| **Partial cure** | 34 (41.0) |
| **Adaptation to microbiology results** | 11 (13.3) |
| **Occurrence of an adverse event leading to stop of C/T** | 3 (3.6) |
| **Treatment failure** | 5 (6.0) |
| **Death** | 2 (2.4) |
| Death linked to the infection | 1 (50.0) |
| **Other reasons for stop** | 4 (4.8) |
| * Unless otherwise specified, values refer to N (%)  1 SD: Standard Deviation |  |
|  |  |
